# *Opuntia* spp.: An Overview of the Bioactive Profile and Food Applications of This Versatile Crop Adapted to Arid Lands

**DOI:** 10.3390/foods12071465

**Published:** 2023-03-29

**Authors:** Carolina Rodrigues, Camila Damásio de Paula, Soufiane Lahbouki, Abdelilah Meddich, Abdelkader Outzourhit, Mohamed Rashad, Luigi Pari, Isabel Coelhoso, Ana Luísa Fernando, Victor G. L. Souza

**Affiliations:** 1 MEtRICs/CubicB, Departamento de Química, NOVA School of Science and Technology, FCT NOVA, Universidade Nova de Lisboa, Campus de Caparica, 2829-516 Caparica, Portugal; 2 Faculdade de Ciências Farmacêuticas, Universidade de São Paulo, São Paulo 05508-900, Brazil; 3 Center of Agrobiotechnology and Bioengineering, Research Unit Labelled CNRST (Centre AgroBiotech-URL-CNRST-05), “Physiology of Abiotic Stresses” Team, Cadi Ayyad University, Marrakech 40000, Morocco; 4 Laboratory of Agro-Food, Biotechnologies and Valorization of Plant Bioresources (AGROBIOVAL), Department of Biology, Faculty of Science Semlalia, Cadi Ayyad University, Marrakesh 40000, Morocco; 5 Laboratory of Nanomaterials for Energy and Environment Physics Department, Faculty of Sciences Semlalia, Cadi Ayyad University, Marrakech 40000, Morocco; 6 Land and Water Technologies Department, Arid Lands Cultivation Research Institute, City of Research and Technological Applications (SRTA-City), New Borg El-Arab City 21934, Alexandria, Egypt; 7 CREA Research Centre for Engineering and Agro-Food Processing, Monterotondo, 00015 Rome, Italy; 8 LAQV-REQUIMTE, Departamento de Química, NOVA School of Science and Technology, FCT NOVA, Universidade NOVA de Lisboa, Campus de Caparica, 2829-516 Caparica, Portugal; 9 INL, International Iberian Nanotechnology Laboratory, 4715-330 Braga, Portugal

**Keywords:** *Opuntia* spp., prickly pear, cladodes, bioactive compounds, food applications

## Abstract

*Opuntia* spp. are crops well adapted to adverse environments and have great economic potential. Their constituents, including fruits, cladodes, and flowers, have a high nutritional value and are rich in value-added compounds. Cladodes have an appreciable content in dietary fiber, as well as bioactive compounds such as kaempferol, quercetin, and isorhamnetin. Fruits are a major source of bioactive compounds such as phenolic acids and vitamin C. The seeds are mainly composed of unsaturated fatty acids and vitamin E. The flowers are also rich in phenolic compounds. Therefore, in addition to their traditional uses, the different plant fractions can be processed to meet multiple applications in the food industry. Several bakery products have been developed with the incorporation of cladode flour. Pectin and mucilage obtained from cladodes can act as edible films and coatings. Fruits, fruit extracts, and fruit by-products have been mixed into food products, increasing their antioxidant capacity and extending their shelf life. Betalains, obtained from fruits, can be used as food colorants and demonstrate promising applications as a sensor in food packaging. This work reviews the most valuable components of the different fractions of this plant and emphasizes its most recent food applications, demonstrating its outstanding value.

## 1. Introduction

Prickly pear is a xerophytic plant belonging to the *Cactaceae* family [1]. This family comprises about 2000 species belonging to 130 genera [2]. *Opuntia* is among the most important and widely distributed genera of the family *Cactaceae* [3]. It is a native plant of arid and semi-arid lands owing to its particular adaptation mechanism to hostile climatic conditions [4]. It is originally from tropical and subtropical regions of America [5]. The species were recognized in Europe as early as the 15th century [6,7]. Afterwards, the cactus was introduced in North Africa, essentially in Morocco, Tunisia, and Algeria, around the 16th century [8]. It has been cultivated later in South Africa, Madagascar, India, Australia, Canada, Brazil, and Argentina [6]. Yet, the cultivation of the cactus is not limited to those areas, being currently present in more than 30 countries due to its importance and multiple applications [6], which are reflected in its environmental, nutritional, and economic benefits [9,10,11].

From an environmental point of view, cactus cladodes provide effective means to fight against desertification, erosion, and soil-related problems [12,13]. They have an exceptional capacity to adapt to drought, thanks to their ability to preserve water in the parenchyma through their specialized photosystem called Crassulacean Acid Metabolism (CAM) [5,14,15,16].

In addition to their environmental importance, this valuable plant is also of great nutritional interest. The cactus fruit is considered a nutritionally complete food, which can also be described as a nutraceutical food [17,18]. It is known for its very high nutritional value in vitamins, mainly ascorbic acid, and minerals, such as magnesium (Mg), calcium (Ca), and potassium (K), as well as being rich in antioxidants like phenolic compounds and flavonoids [19,20,21]. Typically, the cactus fruit is consumed fresh, yet it can be variously formulated into jams, drinks, tea, tinctures, and dietary supplements [22,23,24]. The cladodes have multiple applications as food and feed, most commonly consumed as nutritious fresh vegetables, processed into juice, or bread from their flour [19,25].

Several studies showed that the cactus could be used as a good source of bioactive compounds. due to their richness in secondary metabolites, especially phenolic compounds [25,26]. Among the reported compounds, eucomic acid, kaempferol 3-*O*-robinobioside-7-*O*-arabinofuranoside, isorhamnetin 3-*O*-galactoside, and isorhamnetin 3-*O*-rhamnoside-7-*O*-(rhamnosyl-hexoside) are those with high antioxidant activities [27]. In addition, different health benefits have been associated with cactus consumption and the richness of bioactive compounds, e.g., diabetes prevention [23,28], hypercholesterolemia [29,30], obesity [31], and hypertension [28].

*Opuntia* spp. plants can be used in full, as all vegetative parts are edible (cladodes, fruits, seeds, and flowers). Therefore, this paper represents a bibliographic review of the bioactive composition of prickly pear plants (cladodes, fruits/seeds, flowers, and roots). As knowledge on the extraction procedures of the bioactive compounds of the *Opuntia* plant and its fractions is not commonly reviewed, providing highlights and main findings from multiple studies was also a goal of this work. As this plant is gaining more attention due to its adaptability to marginal soils and arid conditions, which is increasing the area of cultivation and the yearly production, postharvest treatments and different processing options are also being implemented to increase its shelf life and maintain its quality. Hence, recent and innovative food applications of this valuable crop are also addressed in this analysis, with a focus on the full use of this crop, that is, by including examples of application of by-products, following a circular bioeconomy approach.

## 2. Prickly Pear Plant as a Source of Bioactive Macromolecules

### 2.1. Cladodes

Cladodes from *Opuntia* spp. are mainly used as food; i.e., in Mexico, the younger cladodes, called nopalitos, are consumed as fresh vegetables in several dishes or transformed into several food products, foraged, and used as ornaments [9]. Moreover, they find applications in building construction, cosmetics, medicine, and wastewater treatment [32,33].

Cladodes have a high nutritional value due to their minerals, dietary fiber, and phytochemical content [34]. The chemical composition of cladodes varies depending on the type of cultivar, species, maturity stage, environmental conditions, harvesting and post-harvesting conditions, and treatments [27]. Cladodes are mainly composed of water (80–95%), carbohydrates (3–7%), and fiber (1–2%), with low contents of protein and lipids [27,34,35,36]. Furthermore, the pads are a great source of minerals, with a major presence of calcium and potassium and minor quantities of magnesium, manganese, iron, zinc, and copper [37]. The dietary fiber content in cladodes is composed of cellulose, hemicellulose, pectin, lignin, and mucilage [38]. The ingestion of cladodes due to their dietary fiber content may help in reducing body weight, playing an important role in the excretion of lipids [39,40]. Vitamins are also present in cladodes, mainly in the form of ascorbic acid and carotenoids [41,42].

Cladodes are an important source of bioactive compounds such as phenolic compounds (phenolic acids and flavonoids) and carotenoids [43,44]. The most common phenolic compounds found are kaempferol, quercetin, isorhamnetin, and isorhamnetin glucosides [34].

Several studies have been performed using different extraction procedures to access the phytochemical profile of cladodes; the most recent research is summarized in Table 1.

In *Opuntia ficus-indica*, it was possible to identify several bioactive compounds, such as kaempferol, quercetin, and isorhamnetin glucosides [47,48,49,50]. Ben Lataief and collaborators performed both aqueous and ethanolic extractions on *Opuntia dilenii* cladodes to evaluate the differences in the composition of the compounds [46]. Ethanolic extracts allowed the detection of more phenolic and volatile compounds than the aqueous extract. The solvent choice displays an influence on the extraction efficiency and has an impact on the properties and composition of the resulting extracts [51]. Another important factor for bioactive compound extraction from cladodes is the high content of dietary fiber, which can retain those compounds and is where most of them are linked, so an appropriate solvent choice and extraction procedure are recommended [38].

Along with the solvent extraction process for bioactive compounds, new techniques are emerging, as in the case of enzyme-assisted extraction combined with supercritical carbon dioxide extraction. The use of supercritical carbon dioxide and enzymatic hydrolysis proved effective in the isolation of isorhamnetin conjugates [49]. The use of enzymes allowed the accessibility of the phenolic compounds through the breakage of the dietary fiber compounds that are eliminated by the action of CO_2_ and co-solvent during the extraction [52].

The high content of phenols, flavonoids, and also ascorbic acid in cladodes is highly related to several biological effects, such as antioxidant, antibacterial, antifungal, and cytotoxic activities [46,53].

As previously mentioned, cladodes have in their composition an hydrocolloid substance constituted by a complex polysaccharide of high molecular weight, named mucilage, and that substance is produced in specialized plant cells [32,54]. Mucilage represents about 14% of the cladode dry weight, and its physiological function is to regulate the cellular water content during prolonged drought and the calcium fluxes of the plant [35,55]. It is reported that cladodes in an older maturity stage have a decrease in mucilage content because, as a part of the soluble fiber, its content decreases along with the cladode maturation [32]. The mucilage is a complex polysaccharide resulting from the polymerization of monosaccharides like arabinose, galactose, rhamnose, xylose, and uronic acids (e.g., galacturonic acid) and is present in the internal layer of cladodes [56]. The polysaccharide allows the plant to retain a large amount of water and has several functional properties like gelling, thickening, and emulsifying [57]. The mucilage structure is composed of two different water-soluble fractions: pectin with gelling properties with Ca^2+^ and mucilage without gelling properties [58].

Despite the nutritional value of these bio-macromolecules, they are of good interest to the food industry due to their versatility, gelling, and film-forming abilities. Thus, the extraction of mucilage and pectin is comprised of these general steps: removing the outer layers of cladodes to eliminate the spines and the peel; washing and cutting; mixing with a solvent; pressing/centrifugation; precipitation; and drying [59]. Table 2 summarizes the most recent research on the extraction of mucilage and pectin from cladodes.

Conventional methods are used to extract mucilage and pectin from *Opuntia* cladodes, based on the extraction with solvents to isolate those compounds that are all mixed and compose dietary fiber [54,60,61]. The solvents to be used in the extraction and precipitation can be ethanol, methanol, isopropyl alcohol, acetone, or a combination of solvents and can influence the extraction yield, so it is important to determine the best option [70].

Other techniques employed to improve the extraction are the use of ultrasounds, microwave irradiation, and enzyme-assisted extraction [62,63,65]. It is also reported by several authors that acidic, neutral, or basic environments are possible to use in the extraction [66].

The use of ultrasounds allows the reduction of the particle size by the enhancement of the surface area and mass transfer [71]. In microwave-assisted extraction, the irradiation power helps with the diffusion of the solvent into the plant matrix by dissolving the compounds aimed to be extracted [72]. These techniques are helpful in the extraction process by enhancing the extraction efficiency and reducing the use of solvents and the time of extraction [63]. The use of chelating agents such as EDTA that interact with Ca^2+^ also helps with the extraction and improves the process [69]. Since pectins present in cladodes are categorized as low methoxyl pectins, the use of chelating agents for calcium sequestration allows the decrease of the degree of methoxylation by improving the gelling capacity of this pectin [73].

Low-methoxyl pectins have a large application in the food industry due to their gelling and stabilizing properties [34,39,60]. Mucilage can be used as a functional additive and has applications in different industries: as a water purifying agent, as an organic adhesive to lime in construction, as an inhibitor of the corrosion of aluminum, and in the food industry as an edible coating in fruits, while also performing as a stabilizer of emulsions, foams, and suspensions [32,74]. One of the novel potential applications of mucilage is to be used as a material for alternative food packaging once it can replace fossil-based plastics or reinforce polymeric matrices [75].

### 2.2. Fruits

Prickly pears are the succulent fruits from *Opuntia* spp., characterized by their high content in water (almost 92% wt), followed by carbohydrates (4–6% wt), proteins (1–2% wt), and minerals (around 1% wt), of which calcium, phosphorus, and sodium are highlighted [76]. 

*Opuntia* spp. fruits are also a good source of bioactive compounds, especially phenolic compounds, and vitamins (A and C) (Table 3). Their composition depends on several factors, such as soil, place of planting, environmental conditions, age, and species [74], which explains the differences observed in the data found in the literature.

Regarding ascorbic acid (vitamin C), *Opuntia* spp. is rich in this compound due to the light intensity of the planting site. It may also be related to less irrigation and lower temperatures [81]. Regarding the mineral content of *Opuntia ficus-indica*, the values obtained were, considering mg/100 g, 63.4 Mg; 18.7 Na; 108.8 K; 316.5 Ca; 37.8 Mn; 25.9 Fe; 12.6 Zn; 0.01 Cu; and 0.05 P [81]. Thus, it is noticeable that fruits have a significant amount of minerals, in addition to vitamin C, making them advantageous for use as a food supplement [81]. Considering the pulp of the prickly pears, they are rich in biologically active compounds, such as vitamins, polyphenols, carotenoids, and betalains, among others, that can be extracted and used by the pharmaceutical and food industries [78]. It was found that the red-skinned fruit had a total content of phenolic compounds between 164.6 and 218.8 mg per 100 g. There is a large amount of quercetin, isorhamnetin, luteolin, and kaempferol, and it has a relevant content of flavonoids with higher concentrations than edible parts of papaya, banana, and watermelon, for example. Numerous polyphenolic acids such as ferulic acid, p-coumaric acid, 4-hydroxybenzoic acid, caffeic acid, salicylic acid, and gallic acid have also been identified [82]. Peels are also rich in phytochemicals and have a high potential to serve as functional compounds, e.g., in active food packaging films, as was observed with cranberry extracts [83], seaweed extracts [84], different essential oils [85], and plant extracts [86]. The main compounds in the peels are cellulose, hemicellulose, pectin, proteins, antioxidants, flavonoids, minerals, and other polysaccharides [82].

Moreover, their pH, taste, and color are other interesting characteristics that arouse interest for this fruit to be used as food, in addition to the absence of lead and cadmium, which brings greater safety for their consumption [81].

Yet, the amount of by-products reaches around 30% of the total weight after processing the fruits, making it feasible to look for ways to use these by-products in a circular bio-economy approach [77].

According to the study by Elsy De Santiago et al. (2018) [87], cacti have a significant amount of fiber, such as pectin, lignin, mucilage, cellulose, and hemicellulose, which help in the metabolism of glucose and lipids [87].

In addition to the antioxidant properties, other actions are also attributed to the phenolic compounds, namely, anti-inflammatory, anti-diabetes, and anti-cancer [77]. In the composition of these fruits, betalain pigments are also present, which present a red-violet (betacyanins) and yellow-orange (betaxanthins) color. This pigmentation is stable at a pH between 3 and 7, making it possible to use it as a natural color in food and as nutraceuticals, for example [77]. In the study by Tomás García-Cayuela et al. [79], the composition of betalains and phenolic compounds of the peels, pulps, and whole fruit was analyzed and quantified through the evaluation of three varieties from Spain and three from Mexico. The study made a complete comparison of the amounts of these compounds in *Opuntia ficus-indica*. In addition, betalains are natural pigments with active properties (antioxidant, antimicrobial), sensitivity to pH, and other interesting features that could be applied as a bio-based sensor for smart packaging systems. As these compounds are more pH-stable than anthocyanins, their use in smart packaging constitutes a promising alternative [88]. Tests made with the incorporation of amaranth leaf extracts, rich in betalains, in bio-based polymers support this application. In the mentioned study, following the degradation of poultry meat and fish, total volatile basic nitrogen content increased and pH was altered, modifying the betalain chemical structure, which changed the film’s color from red to yellow [89].

The seeds have a high number of compounds beneficial to health, such as unsaturated fatty acids, phytosterols, fat-soluble vitamins (vitamin E), and β-carotene, among others with antioxidant values. They can be used in the food and cosmetics industries and also for the prevention of chronic diseases [82].

The extraction of oil from seeds is traditionally performed using the solid-liquid extraction method with organic solvents such as hexane, chloroform, and petroleum ethers. With this procedure, about 13% of the seed oil can be extracted, depending on the species and the extraction conditions used. This extracted oil is rich in unsaturated acids such as oleic, vaccenic, and linolenic acids. They also have a significant number of tocopherols and phenolic compounds that have antioxidant activity. The total phenolic acid contents in the seed ethanol extract range from 48 to 89 mg in 100 g; and the contents of total flavonoids vary from 1.55 to 2.64 mg in 100 g; the total tannin contents vary between 4.1 and 6.6 mg in 100 g [82].

### 2.3. Opuntia Flowers and Roots

The flowers of *Opuntia* spp. are considered a vegetable, and the fruits and the young cladodes (or nopalitos) can be eaten as such [90]. Moreover, flowers from different plants are known to have wide medicinal properties and are being recognized for their antioxidant properties; however, few data are reported in the literature regarding the phytochemicals and antioxidant properties of *Opuntia* spp. flowers [51]. Traditionally, the flowers from these *Cactaceae* are used for medical purposes; in Tunisian markets, dried flowers of prickly pear are sold and used as an infusion to treat kidney stones [91]. The data found in recent literature on the phytochemical composition and the extraction procedures from *Opuntia* spp. flowers and roots are summarized in Table 4. 

It is noticeable that the flowers and roots of *Opuntia* spp. are rich in phenolic compounds with proven antioxidant and antimicrobial activities [92,93,95]. Once those parts of this perennial crop are generally neglected (i.e., lost in the cultivation process), there are opportunities to prepare such extracts for use either as food additives, with further purification in the pharmaceutical industry, or even as food supplements [94,95]. 

## 3. Applications in Food Products

### 3.1. Food Applications of Cladodes

**Figure 1:**
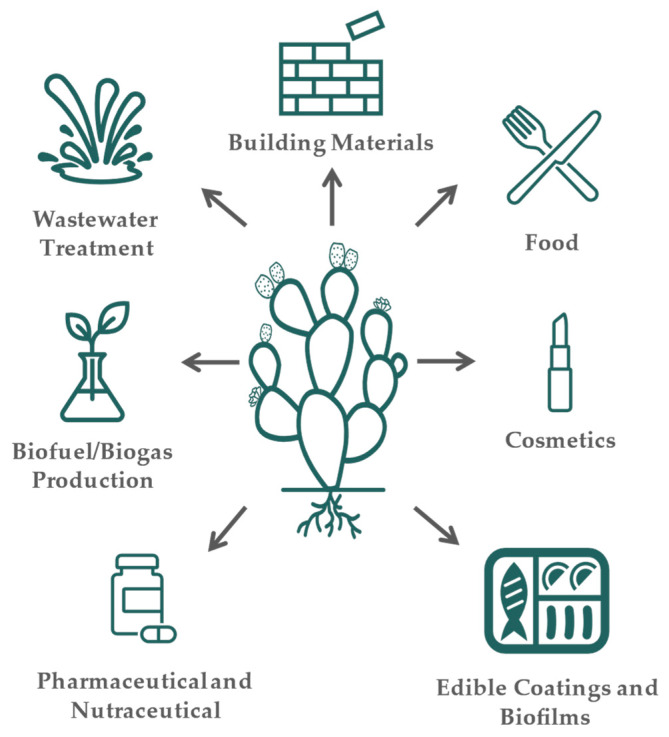
Cladode applications.

Cladodes and their by-products can be used in a variety of industries (Figure 1). The most common uses of cladodes are food and feed consumption [44]. As food, it is consumed fresh or processed into several products such as soups, salads, juices, or bakery products to produce cookies, bread, and biscuits [45,96]. Due to the presence of several compounds with bioactive properties and a high content of dietary fiber, cladodes are also widely applied in cosmetics, pharmaceuticals, and nutraceutical products. As an example, the incorporation of cladodes (up to 10% *w*/*w*) into durum wheat bread revealed an improvement in the antioxidant activity of the bread without affecting the rheological properties [97]. Fortification of pasta with cladodes extracts in substitution of water demonstrated to be useful by increasing the fiber content with antioxidant features and with satisfactory acceptance in sensory analysis [98]. Furthermore, the use of cladodes powder (*Opuntia ficus-indica* f. *inermis*) as a substitute for wheat flour in cookies demonstrated an increase in dietary fiber and mineral content. The cookies produced contained a high level of fat, which makes the cookies highly susceptible to oxidation, but the use of cladodes powder showed a positive effect on reducing oxidation when compared with the control cookies, which were only produced with wheat flour [99]. Cladodes were also added to maize flour to improve the nutritional and physicochemical properties of tortillas in Mexico. Once again, the substitution of maize flour with cladodes powder at 6% improved the dietary fiber and mineral content (e.g., calcium), becoming a source for the intake of these nutrients [100]. In semi-arid regions, cladodes are commonly used for animal feed due to their richness in water and carbohydrates, necessary for their survival [101]. Cladodes were used as whole or supplementary foods, e.g., sheep and goats [102,103].

Mucilage and pectins are also used in food packaging applications as edible coatings and biobased films [104,105]. Some studies showed that *Opuntia ficus-indica* mucilage is effective as a coating material to extend the shelf life of fresh strawberries [106], kiwifruit slices [107], and fig fruit [108]. Edible films produced from mucilage and pectin showed poor mechanical and physical properties, suggesting the need to incorporate other compounds to enhance those characteristics [75,109]. The incorporation of reinforcements, such as nanocellulose, nanoclays, and nanometal oxides, is an option, as it was observed with other biobased polymers (e.g., chitosan) [110,111]. Furthermore, the presence of bioactive compounds on cladodes makes them useful to enhance bioactive characteristics when incorporated in films, e.g., starch-based films, when compared to standalone films [112]. Several additives to improve cactus mucilage film’s mechanical resistance have been studied, e.g., calcium and gelatin [109,113] or beeswax to reduce water vapor permeability [113]. The use of different plasticizers (glycerol, sorbitol, PEG-200, and PEG-400) was also tested, showing that their structural features improved distinct interactions with mucilage polysaccharides [61]. Incorporating essential oils or extracts rich in phenolic compounds can add to the biobased films’ antimicrobial and antioxidant activities, as was observed in other works with biobased polymers [114].

Although more studies are needed to improve the characteristics of the materials, cladodes mucilage and pectins demonstrate promising applications as an alternative to fossil-based plastics currently used in the food industry.

### 3.2. Food Applications of Prickly Pear Fruits

The prickly pear and its by-products can be used in a variety of industries. Various products have been developed with cactus pear residues, such as yoghurts, snacks, and margarine.

In the food industry, one of the uses is the production of juices. To maintain stability and extend their shelf life, heat treatment is commonly used. However, exposure to high temperatures can cause the degradation of thermolabile compounds and modification of the organoleptic temperatures, so alternative technologies have been attempted such as the use of high pressure, pulsed electric fields (PEF), and ultrasound. The presence of phenolic compounds, vitamins, and other bioactive compounds in the juices can be a complement for the consumer [82]. In this sense, the use of PEF technology was applied to help in the inhibition of *S. cerevisiae,* along with pH reduction and the use of preservatives (sodium benzoate and potassium sorbate) in prickly pear juice [115]. The combination of these factors leads to microbial reduction and preservation for 21 days at 25 °C.

Prickly pear fruit is rich in several bioactive compounds, so the addition of this fruit to other food products can provide or enhance several properties, namely, antioxidant activity. That is the case with the incorporation of prickly pear into a gluten-free pasta made from rice-field bean flour, which allowed the increment of phenolic compounds and antioxidant properties at a percentage of 15% (*w*/*w*) [116]. This food product emerges as an alternative for celiac patients. Studies made on consumer preferences in Italy related to using *Opuntia ficus-indica* as an ingredient in new functional pasta showed a significant respondent interest regarding the health benefits and the nutritional and environmental aspects of this type of functional pasta [117]. However, the studies also reveal that the functional pasta should retain the organoleptic and physical properties of durum wheat-based pasta [118]. It was also demonstrated that the introduction of prickly pear peel powder (5% *w*/*w*) in cracker formulation could be a source of dietary fiber and bioactive compounds without affecting the quality of the product. The crackers presented an increment of around 8% in terms of antioxidant activity, and total dietary fiber went from 5.89 g/100 g to 8.11 g/100 g, when compared with the control [119].

Food supplements have been developed by using food by-products from *Opuntia*. Tablets were developed from *Opuntia ficus-indica* L. Mill fruits (green and red varieties) [120]. In this work, the formulation of tablets included the conjugation of microcrystalline cellulose with lactose and the addition of talcum powder and magnesium stearate. Each tablet presented a total weight of between 0.7 and 1 g. In terms of dietary fiber, the tablets presented a content of 0.24 g using green fruit and 0.15 g using red fruit. Furthermore, the tablets showed good antioxidant activity, with DPPH radical inhibition of 24% for the tablets made from red fruits and 20% for green fruits. According to this study, by-products of *O. ficus-indica* have a high potential for use in foods due to their high dietary fiber content and antioxidant activity, which can prevent free radical damage [120].

The use of hydro-ethanolic extracts from prickly pear peels was tested as an alternative to vitamin E in the prevention of margarine oxidation [121]. The extracts produced were rich in phenolic compounds, with a content of 1512.58 mg GAE/100 g of dry matter. Three different concentrations of the extracts were tested (50, 100, and 150 ppm). The use of prickly pear extract in margarine showed a positive effect on the reduction of oxidation when compared with the same product with vitamin E, even at the lowest concentration used (50 ppm). The margarine developed with 150 ppm of extract demonstrated a higher tendency to oxidation, which can be caused by the pro-oxidant effect of the phenolic compounds at high concentrations [121].

Prickly pear extract was added to cooked beef burger patties, and its effect on quality parameters was evaluated [122]. The extract (5% *v*/*w*) was added directly or encapsulated in alginate beads. The encapsulation of bioactive compounds from prickly pear could be a vehicle for their preservation for a long time. The use of prickly pear extracts showed no adverse effects on the cooked burgers. In fact, the intrinsic antioxidant activity present in the extracts, especially the ones encapsulated in alginate, not only enhances that property in the burgers but also avoids lipid oxidation when compared to other burgers in the study.

The seeds present in fruits also have the potential to be used in the food industry due to their richness in fatty acids, in particular, linoleic, palmitic, and stearic acids [123].

### 3.3. Food Applications of Opuntia spp. Flowers

The flowers from *Opuntia* spp. can also be used in food applications, but the most known uses are in decoctions and infusions made from dried flowers, which are widely used in traditional medicine [124]. It is reported that decoctions and infusions from the flowers of *O. ficus-indica* are a source of minerals, namely K and Ca, and also a source of polyphenols, flavonoids, and tannins [125]. The maceration of flowers from *Opuntia ficus-indica* was studied as a heat stabilizer of olive oil as well as its effect on the quality of the final product. The addition of 5% (*w*/*w*) of flowers to the olive oil leads to an increase in the phenolic compound content, improving the stability in terms of oxidation [126].

The knowledge of their characteristics, such as the hydration properties and oil holding capacity, is important as they may interfere with the functionality and nutritional quality of the food [91]. Flowers from *Opuntia ficus-indica* and *Opuntia stricta* harvested from a wild population located in Tunisia in the post-flowering stage were analyzed in terms of their oil holding capacity (OHC) and hydration properties through the determination of the swelling capacity (SWC), water solubility index (WSI), and water holding capacity (WHC). The hydration properties are related to the presence of soluble molecules (such as sugar; more sugar reflects superior WSI), to the polysaccharide content (correlated with SWC), and to the hydrophilic constituents (WHC). The OHC is an important parameter from an industrial point of view as it is correlated to the product’s emulsifying capacity. The results varied depending on the specimen, and overall, *O. ficus-indica* presented superior WSI, WHC, and OHC, while the SWC was higher for *O. stricta.* The values found for SWC were similar to those reported for wheat and carrots but smaller than in cauliflower. Regarding the WHC, *Opuntia* spp. flowers presented values of the same magnitude as other dietary fiber concentrates (from by-products) and some commercial dietary fiber-rich supplements. OHC found were similar to those reported in the cladodes and of the same magnitude as other fruits, vegetables, and seaweeds (around 2 g/g), but lower than for cereal fibers (2–4 g/g) [91].

Thus, *Opuntia* spp. flowers can be highlighted as an excellent alternative source of dietary fiber for human consumption but also as functional ingredients in the food industry as jellying agents to retard syneresis, modify the viscosity and texture of formulated foods, or stabilize food emulsions [91]. Together with the nutraceutical and pharmacological approaches, the use of *Opuntia* spp. flowers can always be explored, adding some economic value to these valuable by-products.

## 4. Conclusions

*Opuntia* spp. is a crop that has been gaining attention throughout the years and has been increasingly studied. Due to its adaptability to adverse environments, it has a high potential to generate value-added products from its fruits and cladodes. Cladodes are a good source of dietary fiber and rich in water, so their consumption should be more considered in the human diet due to their health benefits. Fruits, due to their richness in sugars, can easily be used in the production of juices, jams, and marmalades. Recent studies have used innovative technology to produce food products with greater stability and safety. Moreover, cladodes and fruits are rich in several bioactive compounds and have a high potential to be used in several nutraceutical products. Betalains, which are present in fruits, can also be used as food colorants as an alternative to the ones currently on the market and have a great potential to be used as a sensor in food packaging.

Nevertheless, *Opuntia* spp. by-products and their use in the food industry can be further investigated to better understand the potential uses of this crop in order to enhance its consumption globally.

## Figures and Tables

**Table 1:** Bioactive composition and extraction procedures of *Opuntia* spp. cladodes.

Species/Variety	Extraction Procedure	Main Compounds Found	Conclusions	Ref.
*Opuntia ficus-indica* f. *inermis*	Cladodes were washed, cut, dried, ground, and sieved. Maceration was performed with ethanol. The solvent was evaporated under vacuum. Theextract was dissolved in methanol and properly kept in Liquid Cromatography-mass spectrometry (LC-MS) vials until further analysis.	Quercetin Quercetin 3-*O*-glucoside Kaempferol Kaempferol 3-*O*-glucoside Kaempferol 3-*O*-rutinoside Isorhamnetin Isorhamnetin 3-*O*-glucoside Isorhamnetin 3-*O*-glucoside Isorhamnetin 3-*O*-neohesperidoside 3,3′,4′,5,7-Pentahydroxy-flavanone p-Coumaric acid Zataroside-A Indicaxanthin β-Sitosterol	The analysis of cladode extracts allowed the identification of 13 bioactive compounds, several of them with antioxidant potential.	[45]
*Opuntia dillenii*	Aqueous extract: Extraction with water (24 h, room temperature). Macerates were filtered, lyophilized, and kept at −20 °C.	Quinic acid Protocatechuic acid Caffeic acid Syringic acid p-coumaric acid Naringin Trans ferulic acid Cinnamic acid	Both aqueous and ethanolic cladode extracts presented a good polyphenol profile, with the ethanolic cladode extract presenting highest variability of these bioactive compounds. The aqueous cladode extract was poor in flavonoid compounds.	[46]
	Ethanolic extract: Extraction with ethanol (24 h, room temperature). The macerates were filtered and evaporated in a vacuum rotary evaporator and kept below −20 °C.	Quinic acid Gallic acid Protocatechuic acid Rutin Hyperoside p-coumaric acid Naringin Quercetrin 1,3-di-O-caffeoylquinic acid Apegenin-7-O-glucoside Trans ferulic acid Salviolonic acid Quercetin Kampherol Naringenin Apeginin Luteolin Cirsiliol Cirsilineol Acacetin		
*Opuntia ficus-indica* (L.) Mill	Cladode powder was extracted in an ultrasonic bath with a mixture of methanol/water 80:20 *v*/*v*. Then it was centrifuged, diluted, and filtered.	Piscidic acid I Piscidic acid II 2 Ferulic acid derivative I Eucomic acid Ferulic acid derivative II Kaempferol derivative I Isorhamnetin derivative I Isorhamnetin derivative II Kaempferol derivative II Isorhamnetin derivative III Isorhamnetin 3-O rutinoside (a narcissin)	The analysis of cladode extracts through HPLC-DAD allowed the identification of 11 classes of bioactive compounds. The authors highlighted the presence of piscidic acid (a polyphenol) and isorhamnetin (a flavonoid).	[47]
*Opuntia ficus-indica* (L.) Mill	Extraction was performed in an Ultra-turrax with a mixture of methanol/water 80:20 *v*/*v* and 0.1% formic acid. The resulting extracts were centrifuged, filtered, and finally collected until analysis.	Cyanidin-Glu Pelargonidin-Glu Petunidin-Glu Delphinidin-Glu Malvidin-Glu Luteolin-Glu Apigenin-Glu Isoflavonoids Myricetin-Glu Quercetin-Glu Kaempferol-Glu Isorhamnetin-Glu Furofurans Dibenzylbutyrolactone Alkylphenols Hydroxybenzaldehydes Hydroxycoumarins Tyrosols Hydroxybenzoics Hydroxyphenylpropanoics Hydroxycinnamics	The analysis of cladode extracts through UHPLC-ESI-QTOF-MS allowed the identification of the principal phenolic classes and subclasses. The authors highlighted that cladodes are a good source of anthocyanins and phenolic acids, thus a good source of antioxidant compounds.	[48]
*Opuntia ficus-indica* (L.) Mill	Cladode powder samples were mixed with the enzymes Rapidase or Viscozyme in a mixture of ethanol /water (90:10). The vessel was subjected to pressure with CO_2_, and then a supercritical fluid extraction was performed.	3-*O*-methyl quercetin 3-*O*-methyl kaempferol Luteolin Isorhamnetin tri- and diglycosides: Isorhamnetin-3-*O*-glucosyl-rhamnosyl-rhamnoside Isorhamnetin-3-*O*-glucosyl-rhamnosyl-pentoside Isorhamnetin-3-*O*-glucosyl-rhamnosyl-methylpentoside Isorhamnetin-3-*O*-glucosyl-pentoside Isorhamnetin-3-*O*-glucosyl-rhamnoside	The use of enzymes to assist the extraction with supercritical fluids improved the extraction of isorhamnetin conjugates.	[49]
*Opuntia ficus-indica* (L.) Mill	Cladodes powder was subjected to sequential extractions with different solvents. The resulting supernatants were mixed, and acid hydrolysis was performed under reflux using different concentrations of HCl and times.	Isorhamnetin Kaempferol Quercetin Total flavonoids Ferulic acid 4-Hydroxybenzoic acid	Acid hydrolysis was demonstrated to be advantageous for identifying and quantifying (poly)phenolic compounds through High Performance Liquid Chromatography with Diodde Array Detection (HPLC-DAD). The highest amount of total (poly)phenolic compounds was obtained from acid hydrolysis under the following conditions: 2 h, 90 °C, 1.5 M HCl.	[50]

**Table 2:** Mucilage and pectin extraction procedures, yields, and compositions from cladodes of *Opuntia* spp.

Species/Variety	Extraction Procedure	Yield of Extraction (%)	Main Compounds Found	Conclusions	Ref.
*Opuntia ficus-indica*	Cladode powder was subjected to a sequential extraction to obtain water-soluble pectins (WSP), chelating-soluble pectins (CSP), and acid-soluble pectins (ASP).	WSP: 5.75% (*w*/*w*) of dry weight CSP: 0.21% (*w*/*w*) of dry weight ASP: 0.11% (*w*/*w*) of dry weight	Monosaccharide composition: Galactose Glucose Galacturonic acid Arabinose Xylose Rhamnose Mannose	All the pectin fractions obtained are mostly rich in galacturonic acid. Through the degree of methylation analysis, it was shown that all pectin fractions are lowly methylated (DM < 50%).	[60]
*Opuntia ficus-indica*	Mucilage extraction: Cladodes were washed and cut, and the interior was removed and pressed. Mucilage was precipitated in ethanol in a ratio of 2:3 (extract: ethanol). The precipitate was collected, washed with ethanol, dried (50 °C, 24 h), and then pulverized.	Not presented	Monosaccharide composition: Galactose Arabinose Xylose Rhamnose Glucose	The major sugars present in cactus mucilage are galactose and arabinose.	[61]
*Opuntia monocantha*	Mucilage extraction: Cladodes were washed, cut, and crushed. The resulting pulp was mixed with water at a ratio of 1:2 *w*/*v* (pulp: water), stirred, and heated (80 °C, 30 min). The mixture was filtered and centrifuged. The supernatant was precipitated in 95% ethanol and kept overnight at 4 °C. The mucilage was filtered under vacuum, dried, and sieved.	12.0% (*w*/*w*) of dry weight	Monosaccharide composition: Arabinose Galactose Glucose Rhamnose Xylose Galacturonic acid Glucuronic acid	The extraction method shows promising results for obtaining mucilage powder, which is mainly composed of carbohydrates.	[54]
*Opuntia ficus-indica*	Total pectic mucilage fraction extraction (TFC): Extraction is performed in a water bath using water as the solvent (adjusting the pH). The mixture was filtered and centrifuged, and the supernatant was concentrated. The solution was precipitated in isopropanol (4 °C, overnight). The resulting precipitate was centrifuged, washed with ethanol, and dried. Mucilage fraction extraction (MC): The extraction procedure was performed under agitation with water as the solvent and centrifuged. The supernatant was precipitated with isopropanol (4 °C, overnight), then washed with absolute ethanol and dried. Pectin fraction extraction (PC): obtained by the same method used for total pectic mucilage fraction; using the residues from the mucilage extraction as starting materials.	TFC: 13.1% (*w*/*w*) of dry weight MC: 10.2% (*w*/*w*) of dry weight PC: 6.13% (*w*/*w*) of dry weight	High content in carbohydrates	Extraction conditions may affect the extraction yield. PC was richer in uronic acid content than the other two fractions. Mucilage showed a lower content of uronic acid.	[57]
*Opuntia ficus-indica*	Pectin extraction: extraction from cladode’s powder was made with water, centrifuged, and the resulting residues were dried. The residues were mixed with water in an ultrasound water bath and centrifuged. The supernatant was precipitated in isopropanol and then dried.	18.6% (*w*/*w*) of dry weight	High uronic acid content	The use of ultrasounds increased the pectin extraction and reduced the extraction time when compared with chemical extraction methods.	[62]
*Opuntia ficus-indica*	Mucilage was extracted from cladodes by performing a microwave-assisted extraction (different powers were applied). Then the samples were filtered, centrifuged, and precipitated in ethanol. The resulting precipitate was washed with ethanol and lyophilized.	8.13% (*w*/*w*) of dry weight with 500 W for 7 min	Monosaccharide composition: Galactose Arabinose Xylose Rhamnose Glucose	Microwave irradiation promotes the interaction between the solvent and the material, enhancing the extraction.	[63]
*Opuntia dillenii* (Ker-Gawl) Haw	Mucilage extraction: fresh cladodes were extracted with water under stirring. The solution was filtered and concentrated. The concentrated solution was precipitated with and dialyzed against water (24 h, membrane of 12–14 kDa) and lyophilized.	6.20% (*w*/*w*) of dry weight	Monosaccharide composition: Arabinose Galactose Rhamnose Xylose Glucose Uronic acid	The major neutral sugars found were arabinose and galactose. The uronic acid was found in a lower percentage (2.5%), demonstrating the neutrality of the mucilage.	[64]
*Opuntia ficus-indica*	Mucilage was previously extracted from cladodes, and the resulting residue was used for pectin enzyme-assisted extraction. Xylanase and cellulase were added under optimal conditions. After enzyme inactivation, the mixture was centrifuged, and the supernatant was precipitated with isopropanol. The precipitate was centrifuged, dried at 50 °C, and reduced to powder.	16.7% (*w*/*w*) of dry weight	Arabinose Xylose Fructose Glutamic acid Galacturonic acid Glucose Galactose Mannose Rhamnose	Enzyme-assisted extraction proved effective for enhancing pectin extraction without using acid treatments. The resulting pectin was lowly methylated and showed a galacturonic acid content of 65%.	[65]
*Opuntia ficus-indica*	Pectin extraction was performed under three different extraction conditions: acidic (pH = 2), neutral (pH = 6), and basic (pH = 10). The samples were submitted to ultrasound and then placed under mechanical stirring, filtered, and centrifuged. The residues were submitted to the same extraction, and the supernatant was mixed at the end. The resulting supernatants were precipitated in ethanol and filtered. The residues were dialyzed (30 kDa membrane) against water, concentrated, and lyophilized.	Acidic (pH = 2): 8.80% (*w*/*w*) of dry weight Neutral (pH = 6): 24.5% (*w*/*w*) of dry weight Basic (pH = 10): 4.60% (*w*/*w*) of dry weight	Neutral sugars Galacturonic acid	Neutral extraction obtained the highest total yield, although acidic extraction presented a higher galacturonic acid content.	[66]
*Opuntia ficus-indica*	Mucilage extraction was performed by extrusion. The solution was centrifuged, and the supernatant was precipitated in ethanol and dried.	1.50% (*w*/*w*) of dry weight	Not assessed	The yield of extraction was demonstrated to be dependent on mucilage synthesis, which depends on edaphoclimatic conditions.	[67]
*Opuntia spinulifera* Salm-Dyck	Mucilage extraction was accomplished by mixing cladodes with water under stirring and filtering at the end (process repeated four times). The filtrate obtained was precipitated with ethanol, dried, and reduced to powder.	Not assessed	Arabinose Rhamnose Xylose Galactose Uronic acid	Cladodes from *Opuntia spinulifera* are rich in carbohydrates. Furthermore, the presence of pectins was found through Fourier-Transform Infrared Spectroscopy (FTIR) analysis. This could be explained by the ability of pectins to also precipitate in ethanol.	[68]
*Opuntia ficus-indica*	Residue from cladode’s flour was obtained by depigmentation with ethanol and acetone and centrifugation. The extraction was performed by mixing EDTA and water, adjusting the pH, and stirring. Then, a centrifugation was performed, and the supernatant was precipitated. The precipitate was recovered through centrifugation and resuspended in water. The precipitate was filtered, and the solution was mixed with ethanol (5 °C, overnight), filtered again, and then dried.	10.4% (*w*/*w*) of dry weight under conditions of: pH = 11; 20% EDTA; 80 °C	Galacturonic acid Arabinose Galactose Glucose Rhamnose	Pectin extractions under alkaline conditions demonstrated a higher yield than those performed under acidic conditions. The use of EDTA suggests that the chelation of calcium is an important factor in extraction.	[69]

**Table 3:** Bioactive composition and extraction procedures of prickly pears and seed oil.

Species/Variety	Provenance	Extraction Procedure	Main Bioactive Compounds	Main Conclusions	Ref.
*Opuntia ficus-indica* var. Gialla	The fruits were purchased from Sicily, Italy, and Portugal. They were separated according to color; i.e.,orange-red (*Opuntia ficus-indica* var. Gialla) and red-violet (*Opuntia ficus-indica* var. Sanguigna)	Hydro-alcoholic extraction (ethanol:water) was performed under agitation and followed by filtration. The resulting extracts were lyophilized and their content in total phenolic compounds, antioxidants, and betalain, was evaluated by using High Performance Liquid Chromatography (HPLC).	Piscidic acid: 1061 mg/g fruit Eucomic acid: 1.40 mg/g fruit Isorhamnetin- *O*—(di-deoxyhexosylhexoside): 0.22 mg/g fruit Isorhamentin- *O*—(deoxyhexosyl-pentosylhexoside): 0.77 mg/g fruit Isorhamnetin- *O*—(pentosylhexoside): 0.10 mg/g fruit Total phenolic compounds: 3.26 mg/g fruit Betaxanthines—Indicaxanthin I isomer: 25.3 mg/g fruit Indicaxanthin II isomer:64.5 mg/g fruit Betacyanins—Betanidin-5-*O*-*β*- glucoside (betanine): 1.25 mg/g fruit Total: 1.25 mg betacyanin compounds/g fruit	*Opuntia ficus-indica* var. Sanguigna has more phenolic compounds and betacyanin compounds than *Opuntia ficus-indica* var. Gialla	[77]
*Opuntia ficus-indica* var. Sanguigna			Eucomic acid: 2.2 mg/g fruit Isorhamnetin- *O*—(di-deoxyhexosylhexoside): 0.56 mg/g fruit Isorhamnetin- *O*—(di-deoxyhexosylhexoside): 0.21 mg/g fruit Isorhamentin- *O*—(deoxyhexosyl-pentosylhexoside): 1.3 mg/g fruit Isorhamentin- *O*—(pentosylhexoside): 0.67 mg/g fruit Isorhamentin- *O*—(deoxyhexosylhexoside): 1.7 mg/g fruit Total phenolic compounds: 3.7 mg/g fruit Indicaxanthin I isomer: 56.0 mg/g fruit Indicaxanthin II isomer: 2.8 mg/g fruit Betanidin-5-*O* -*β*-glucoside (betanine): 3.44 mg/g fruit Isobetanine: 0.54 mg/g fruit Total beta-cyanine compounds: 3.97 mg/g fruit		
*Opuntia ficus-indica* (green peel)	Fruits were harvested from Texas A&M University, Kingsville orchard. Following that, the separation of the peels and pulp was performed and the material was weighed and stored at −80 °C.	Flavonoid extraction: skin and pulp were homogenized with 25 mL of methanol. Acid hydrolysis was performed with the flavonoids aglycones. Quantification was performed by using HPLC. Ascorbic acid extraction: frozen tissues were sprayed with a dismembrator, centrifuged, and the supernatant was filtered. Quantification was performed by using HPLC. Carotenoids extraction: skin and pulp were mixed with a mixture of hexane/acetone/methanol, followed by centrifugation, and then filtration. Total carotenoids determination was performed by using a UV-Vis spectrophotometer.	Flavonol content Kaempferol: 2.2 µg/g fruit Quercetin: 43.2 µg/g fruit Isorhamnetin: 24.1 µg/g fruit Ascorbic acid: 458 µg/g fruit Total carotenoids: 2.9 µg/g fruit Total flavonoids: 69.5 µg/g fruit	*Opuntia stricta* var. stricta has presented more Kaempferol; *Opuntia streptacantha* has more Quercetin; and the *Opuntia ficus-indica* has more Isorhamnetin. In addition to that, *Opuntia stricta* var. stricta has more ascorbic acid; *Opuntia lindheimeri* has more carotenoids; and *Opuntia streptacantha* has more flavonoids.	[78]
*Opuntia streptacantha* (red peel)			Flavonol content Kaempferol: 1.1 µg/g fruit Quercetin: 90.5 µg/g fruit Isorhamnetin: 1.9 µg/g fruit Ascorbic acid: 121 µg/g fruit Total carotenoids: 6.7 µg/g fruit Total flavonoids: 93.5 µg/g fruit		
*Opuntia stricta* var. stricta (yellow peel)			Flavonol content Kaempferol: 3.8 µg/g fruit Quercetin: 51.0 µg/g fruit Isorhamnetin: not detected Ascorbic acid: 815 µg/g fruit Total carotenoids: 14.6 µg/g fruit Total flavonoids: 54.8 µg/g fruit		
*Opuntia lindheimeri* (purple peel)			Flavonol content Kaempferol: not detected Quercetin: 9.8 µg/g fruit Isorhamnetin: not detected Ascorbic acid: 437 µg/g fruit Total carotenoids: 23.7 µg/g fruit Total flavonoids: 9.8 µg/g fruit		
*Opuntia ficus-indica* (Mexico, purple peel)	The fruits used in this study were obtained from Mexico and Spain. They were chosen based on their similar characteristics in terms of ripeness, uniformity, and quality of the material.	Extraction was performed on freeze-dried fruits using methanol and water. The mixture passed through an ultrasonic bath and was centrifuged. After removing the supernatant, the process was repeated twice. The supernatant was analyzed by HPLC.	Whole fruit Betaxanthins: 246.2 µg/g fruit Betacyanins: 2176 µg/g fruit Betalains: 2423 µg/g fruit Phenolic acids: 27,556 µg/g fruit Flavonoids: 643.4 µg/g fruit Phenolics: 28,199 µg/g fruit Pulp Betaxanthins: 206.8 µg/g fruit Betacyanins: 2070 µg/g fruit Betalains: 2274 µg/g fruit Phenolic acids: 18,498 µg/g fruit Flavonoids: 319.6 µg/g fruit Phenolics: 18,818 µg/g fruit Peel Betaxanthins: 69.1 µg/g fruit Betacyanins: 630 µg/g fruit Betalains: 699 µg/g fruit Phenolic acids: 45,587 µg/g fruit Flavonoids: 3426 µg/g fruit Phenolics: 49,012 µg/g fruit	The whole fruits of the purple-colored species from Mexico and Spain were the richest in betacyanin, and those of red coloration from Spain and yellow from Mexico have a higher content of betaxanthin. Regarding the peel, a large amount of phenolic compounds was observed, mainly in the purple variety from Spain.	[79]
*Opuntia ficus-indica* (Mexico, red peel)			Whole fruit Betaxanthins: 177.9 µg/g fruit Betacyanins: 404.7 µg/g fruit Betalains: 582.6 µg/g fruit Phenolic acids: 23,169 µg/g fruit Flavonoids: 798 µg/g fruit Phenolics: 23,967 µg/g fruit Pulp Betaxanthins: 246.8 µg/g fruit Betacyanins: 525 µg/g fruit Betalains: 771.8 µg/g fruit Phenolic acids: 13,030 µg/g fruit Flavonoids: 171.7 µg/g fruit Phenolics: 13,202 µg/g fruit Peel Betaxanthins: 118.3 µg/g fruit Betacyanins: 389.4 µg/g fruit Betalains:507.6 µg/g fruit Phenolic acids: 42,106 µg/g fruit Flavonoids: 2564 µg/g fruit Phenolics: 44,669 µg/g fruit		
*Opuntia ficus-indica* (Mexico, yellow peel)			Whole fruit Betaxanthins: 487.9 µg/g fruit Betacyanins: 100.1 µg/g fruit Betalains: 587.9 µg/g fruit Phenolic acids: 19,990 µg/g fruit Flavonoids: 1073 µg/g fruit Phenolics: 21,063 µg/g fruit Pulp Betaxanthins: 390.8 µg/g fruit Betacyanins: 89.2 µg/g fruit Betalains: 479.9 µg/g fruit Phenolic acids: 8902 µg/g fruit Flavonoids: 139.5 µg/g fruit Phenolics: 9042 µg/g fruit Peel Betaxanthins: 252.3 µg/g fruit Betacyanins: 96.1 µg/g fruit Betalains: 384.4 µg/g fruit Phenolic acids: 28,074 µg/g fruit Flavonoids: 3218 µg/g fruit Phenolics: 31,292 µg/g fruit		
*Opuntia ficus-indica* (Spain, purple peel)			Whole fruit Betaxanthins: 278.7 µg/g fruit Betacyanins: 1372 µg/g fruit Betalains: 1651 µg/g fruit Phenolic acids: 27,252 µg/g fruit Flavonoids: 615.3 µg/g fruit Phenolics: 27,867 µg/g fruit Pulp Betaxanthins: 209.8 µg/g fruit Betacyanins: 1084 µg/g fruit Betalains: 1293 µg/g fruit Phenolic acids: 23,131 µg/g fruit Flavonoids: 204.0 µg/g fruit Phenolics: 23,335 µg/g fruit Peel Betaxanthins: 175.7 µg/g fruit Betacyanins: 1022 µg/g fruit Betalains: 1197 µg/g fruit Phenolic acids: 35,163 µg/g fruit Flavonoids: 3268 µg/g fruit Phenolics: 38,430 µg/g fruit		
*Opuntia ficus-indica* (Spain, red peel)			Whole fruit Betaxanthins: 435.4 µg/g fruit Betacyanins: 384.4 µg/g fruit Betalains: 819.8 µg/g fruit Phenolic acids: 17,022 µg/g fruit Flavonoids: 550.9 µg/g fruit Phenolics: 17,573 µg/g fruit Pulp Betaxanthins: 405.1 µg/g fruit Betacyanins: 343.0 µg/g fruit Betalains: 1293 µg/g fruit Phenolic acids: 14,340 µg/g fruit Flavonoids: 161.5 µg/g fruit Phenolics: 14,502 µg/g fruit Peel Betaxanthins: 210.3 µg/g fruit Betacyanins: 379.3 µg/g fruit Betalains: 589.6 µg/g fruit Phenolic acids: 36,183 µg/g fruit Flavonoids: 3571 µg/g fruit Phenolics: 39,754 µg/g fruit		
*Opuntia ficus-indica* (Spain, yellow peel)			Whole fruit Betaxanthins: 134.8 µg/g fruit Betacyanins: 21.4 µg/g fruit Betalains: 156.2 µg/g fruit Phenolic acids: 18,165 µg/g fruit Flavonoids: 554.9 µg/g fruit Phenolics: 18,720 µg/g fruit Pulp Betaxanthins: 214.2 µg/g fruit Betacyanins: 26.2 µg/g fruit Betalains: 240.5 µg/g fruit Phenolic acids: 10,437 µg/g fruit Flavonoids: 117.2 µg/g fruit Phenolics: 10,554 µg/g fruit Peel Betaxanthins: 98.0 µg/g fruit Betacyanins: 27.8 µg/g fruit Betalains: 125.7 µg/g fruit Phenolic acids: 38,820 µg/g fruit Flavonoids: 2860 µg/g fruit Phenolics: 41,680 µg/g fruit		
*Opuntia ficus-indica* (green peel)	Fruits were harvested in Bejaia, Argelia, in August 2008. They were selected based on spines, colors, and shapes. This study compared the seed oil composition extracted from *Opuntia ficus-indica* of different peel colors (green, yellow, orange, and red).	Seed oil was obtained by Soxhlet extraction, using hexane. For the extraction of phenolic compounds from the oil, a solid-liquid extraction method was applied using ethanol. The content in phenolic compounds was assessed by the Velioglu method and also analyzed by High Performance Liquid Chromatography -Mass Spectrometry (HPLC-MS).	Total phenolic compounds: 61 mg GAE */100 g seed Flavonoids: 1.5 mg/100 g seed Tannins: 4.5 mg/100 g seed	The orange variety has more phenolic compounds. The study concludes that the seeds from *Opuntia ficus-indica* have a good number of phenolic compounds that can be used by the industry.	[80]
*Opuntia ficus-indica* (yellow peel)			Total phenolic compounds: 74 mg GAE */100 g seed Flavonoids: 1.9 mg/100 g seed Tannins: 4.8 mg/100 g seed		
*Opuntia ficus-indica* orange peel)			Total phenolic compounds: 89 mg GAE */100 g seed Flavonoids: 2.6 mg/100 g seed Tannins: 6.6 mg/100 g seed		
*Opuntia ficus-indica* (red peel)			Total phenolic compounds: 48 mg GAE */100 g seed Flavonoids: 1.5 mg/100 g seed Tannins: 4.1 mg/100 g seed		

* GAE (Gallic Acid Equivalent).

**Table 4:** Bioactive composition and extraction procedures of *Opuntia* spp. flowers and roots.

Species/Variety	Provenance	Extraction Procedure	Main Bioactive Compounds and Levels Found	Main Conclusion	Ref.
*Opuntia ficus-indica*	Flowers were collected during the post-flowering stage in June 2013, from wild populations located in Tunisia (latitude 34°46′29″ N, longitude 10°39′73″ E; elevation: 41 m), where the climate is semi-arid, characterized by a mean rainfall of 200 mm/year.	Extraction procedure was performed by using different solvents: water, methanol, acetonitrile, acetone, ethyl acetate, dichloromethane, and hexane. Two different extraction processes were tested: Soxhlet extraction and maceration.	Total phenolic content (mg GAE */g extract) Soxhlet extraction Water: 58.7 Methanol: 270.9 Acetonitrile: 132.4 Acetone: 67.5 Ethyl acetate: 122.3 Dichloromethane: 10.1 Hexane: 7.5 Maceration extraction Water: 44.2 Methanol: 227.8 Acetonitrile: 17.6 Acetone: 70 Ethyl acetate: 70.8 Dichloromethane: 14.7 Hexane: 14.8 Total flavonoid content (mg RE*/g extract) Soxhlet extraction Water: 9.7 Methanol: 60.81 Acetonitrile: 19.4 Acetone: 15.29 Ethyl acetate: 17.65 Dichloromethane: 1.73 Hexane: Not detected Maceration extraction Water: 22.47 Methanol: 27.47 Acetonitrile: 3.25 Acetone: 4.6 Ethyl acetate: 15.75 Dichloromethane: 8.11 Hexane: Not detected	In terms of extract yield, maceration is more efficient than Soxhlet, and the two best solvents are water and methanol. Regarding the bioactive compounds and antioxidant activity, the solvent’s polarity had a significant effect on antioxidant activity. Aqueous and methanolic extracts have proved to be best in terms of high extraction of phenolic compounds and antioxidant activity. It was proven that the polyphenols are thermostable, and once they are responsible for the antioxidant activity of the flower extracts, the Soxhlet method was the most interesting to preserve the antioxidant activity.	[51]
*Opuntia ficus-indica* and *Opuntia stricta*	From May to June 2009, the flowers were collected from 4 different flowering stages (vegetative, initial flowering, full flowering, and post-flowering) from wild populations located in Tunisia (latitude 34°46′29″ N, longitude 10°39′73″ E; elevation: 41 m), where the climate is semi-arid and is characterized by a mean rainfall of 200 mm/year.	Maceration was used for extraction using hexane. After 7 days, extracts were filtered and concentrated and finally characterized by Gas Chromatography with flame-ionization detection (GC FID).	Fifty different components were detected, representing around 85–99.7% of the total integrated peak area. No remarkable differences were observed between the flower compositions. Phytochemical compounds present in the *Opuntia* flower extract essentially belong to carboxylic acids (28–97%), terpenes (0.2–57%), esters (0.2–27%), and alcohol classes (<1.8%). Aromatic compounds, mostly phenolic compounds ((2-methoxy-4-vinylphenol and phenol) represented only 9.3% of the identified molecules. Monoterpenes d1-limonene and linalool were found in small quantities in the early stages of *O. ficus-indica* flowering, and only at post-flowering for *O. stricta*. Camphor represented 32.4% of the compounds found in the vegetative stage for *O. ficus-indica*. In terms of total phenolic content: *Opuntia ficus-indica*: Vegetative: 16.3 mg GAE */g extract Initial flowering: 13.4 mg GAE */g extract Full flowering: 13.3 mg GAE */g extract Post-flowering: 49.0 mg GAE */g extract *Opuntia stricta*: Vegetative: 16.7 mg GAE */g extract Initial flowering: 15.3 mg GAE */g extract Full flowering: 13.1 mg GAE */g extract Post-flowering: 80.9 mg GAE */g extract	The extraction yield increased with the evolution of the flowering stage, with the highest yield found at the post-flowering stage (*O. ficus-indica*) and for *O. stricta* at full flowering. Overall, *O. stricta* presented more phenolic compounds than *O. ficus-indica*, which corroborates the superior antioxidant activity of its extract. However, the antioxidant power was not related to the number of phenolic compounds; once at the post-flowering stages, it presented the highest content but the lowest activity. This property was attributed to the presence of terpenes and aromatic compounds. The extracts exhibited good antimicrobial activity against *Pseudomonas aeruginosa, Staphylococcus aureus,* and *Escherichia coli*, with *O. stricta* extracts being more active than *O. ficus-indica*.	[92]
*Opuntia ficus-indica* (L) Miller	Flowers were collected during the post-flowering stage in June 2014 from wild populations located in Tunisia (latitude 34°46′29″ N, longitude 10°39′73″ E; elevation: 41 m), where the climate is semi-arid, characterized by a mean rainfall of 200 mm/year.	Two flower extracts were evaluated: an aqueous extract (mucilage) and a methanolic extract (Soxhlet extraction). Mucilage extract was characterized in terms of monosaccharide composition by GC. Furthermore, antimicrobial activity was evaluated against Gram-positive and Gram-negative foodborne bacteria.	Mucilage composition found: Glucose: 32.41 mg/g extract Mannose: 11.46 mg/g extract Arabinose: 7.98 mg/g extract Xylose: 4.97 mg/g extract Galactose: 2.61 mg/g extract Rhamnose: 1.99 mg/g extract Total sugar: 61.4%	The mucilage yield of th extraction found was 18.3%, while 14.8% was reported for the Soxhlet extract. Glucose was the major component of the mucilage and originated from the plant flower’s hemicellulose and cellulose. Both mucilage and methanolic extracts exhibited antimicrobial activity against both Gram-positive and Gram-negative foodborne bacteria, with a superior effect against the former. The methanolic extract was more efficient against the bacteria tested than mucilage, and the highest inhibition was found against *Listeria monocytogenes*. In terms of antioxidant properties, the mucilage and methanol extracts exhibited significant anti-radical activity, although the mucilage extract showed lower radical-scavenging activity than the methanolic extract.	[93]
*Opuntia ficus-indica* (L) Miller	Flowers of *O. ficus-indica* were collected in Monasterace, Italy, in May 2007.	Dried flowers were subjected to a maceration extraction process using methanol. Purification was performed by using Solid-Phase Extraction. The dried residue was analyzed by High Performance Liquid Chromatography coupled with Photo Diode Array and Electrospray Ionization Gas Chromatography (HPLC-PDA-ESI-MS) for qualitative investigation and by HPLC-PDA for quantitative analysis. Volatiles were analyzed by GC and Gas Chromatography with Electron Impact Mass Spectrometry (GC-EIMS) system.	*O. ficus-indica* flower methanol extract composition (mg/g): Quercetin 3-*O*-rutinoside: 7.09 Kaempferol 3-*O*-rutinoside: 4.00 Quercetin 3-*O*-glucoside: 4.47 Isorhamnetin 3-*O*-robinobioside: 42.69 Isorhamnetin 3-*O*-galactoside: 9.79 Isorhamnetin 3-*O*-glucoside: 7.24 Kaempferol 3-*O*-arabinoside: 3.24 Composition of *O. ficus-indica* flower volatiles (%): (E)-3-Hexen-1-ol: 3.7 1-Hexanol: 12.3 Nonanal: 2.5 2-Ethyl hexyl acetate: 1.2 Tetrahydrolavandulol: 5.5 1-Nonanol: 1.2 Pinocampheol: 1.7 Dihydrocitronellol: 2.6 Decanal: 8.2 Tridecane: 5.9 cis-2,3-Pinanediol: 1.9 η-Tetradecane: 9.1 β-Ylangene: 1.5 (E)-Geranyl acetone: 4.8 Allo-aromadendrene: 3.9 Germacrene D: 12.6 η –Pentadecane: 4.0 η –Hexadecane: 1.6	Secondary metabolites belonging to the flavonol glycoside class were found in the cactus flowers. Total flavonoids of the flowers were 81.75 mg/g of fresh plant material, and Isorhamnetin 3-*O*-robinobioside was the major component, representing 52.22%, followed by isorhamnetin 3-*O*-galactoside (11.98%). The flower extract has a pharmaceutical interest as it can be used for the treatment of depression, and its major component is associated with a testosterone 5α-reductase inhibitor. This was the first report on the volatile composition of *O. ficus-indica*. There were no monoterpene hydrocarbons found, but oxygenated monoterpenes were at 16.5% and sesquiterpene hydrocarbons were at 18%, with Germacrene D as the major component.	[94]
*Opuntia ficus-indica* f. *inermis*	The roots were collected from municipal areas of Gafsa, Tunisia.	Extraction from dried roots was performed by using methanol under stirring. The solution was then centrifuged, and the supernatant was dried. Total phenolic compounds and flavonoid content were determined using UV/Vis spectroscopy.	Total phenolic: 57.56 mg GAE */g extract Total flavonoids: 23.5 mg RE*/g extract	The yield of extraction found was 26%. Root methanolic extract exhibited remarkable content in phenolic and flavonoid compounds. In comparison to swallow root (*Decalepis hamiltonii*), *Opuntia* root extract presented almost double the phenolic compounds. Regarding the flavonoid content, *Opuntia* root extracts reported concentrations superior to those reported for *Opuntia* fruit extracts, which are known for their antioxidant power. Extracts presented antiulcerogenic activities tested in vivo in rats. Phenolic and flavonoid wealth, radical scavenging activity, and reducing power have been implicated in the extract’s antiulcer properties.	[95]

* GAE (Gallic Acid Equivalent), RE (Rutin Equivalent).

## Data Availability

Data sharing is not applicable.
